# Quantifying gravity wave forcing using scale invariance

**DOI:** 10.1038/s41467-019-10527-z

**Published:** 2019-06-13

**Authors:** Han-Li Liu

**Affiliations:** High Altitude Observatory, National Center for Atmospheric Research, P.O.Box 3000, Boulder, CO 80307-3000 USA

**Keywords:** Climate sciences, Atmospheric science, Atmospheric dynamics

## Abstract

Despite the increasing resolution, forcing on the mean circulation by resolved waves in general circulation models is not yet converging. Parameterization of the forcing remains a major source of model uncertainty. This study examines the scale invariance of zonal spectra of momentum flux and wave forcing, and shows that it can be used to quantify the forcing by unresolved waves with knowledge of the resolved ones in global models. The result reveals the leading order importance of the small-scale wave forcing, which is in general agreement with that required for obtaining the zonal mean wind climatology. It is also found that wave and mean flow interaction is important in maintaining the robust spectral structure. This method may provide a strategy to design physically consistent and scale-aware parameterization schemes for scale invariant quantities, when a model has sufficient resolution to partially resolve their spectra.

## Abstract (web summary)

Parametrizations of unresolved small-scale atmospheric waves are an important source of uncertainty in climate models. Scale invariance is used to estimate the forcing of these small-scale waves and it is shown to have a leading order effect on the mean flow.

## Introduction

Scale invariance of kinetic energy (KE) spectrum is well established from atmospheric wind observations^1^ and can be reproduced in high-resolution general circulation models (GCMs) over part of the observed scale ranges, as allowed by model resolution^2–7^. Although there are competing theories for the observed ~−5/3 slope of the KE spectrum over the mesoscale range (several hundred km down to ~20 km^8^), high-resolution numerical studies provide support for the interpretation in terms of gravity waves: they have shown that the KE spectrum of the flow divergence modes, presumably associated with gravity waves, follows a ~−5/3 slope and that it becomes increasingly dominant over the rotational modes (following a ~−3 slope) at higher altitudes^2,6,9^. This is consistent with the finding from other GCMs, in particular those with high model tops, that the transition from the −3 slope to the −5/3 slope occurs at larger horizontal scales at higher altitudes, where gravity waves become increasingly dominant^2,3,7,10^. The KE spectrum of the gravity waves may result from forward cascade with excitation by large-scale stirring^11–14,^ from inverse cascade with smaller scale stirring^15–17,^ or from both^18^.

Although the KE spectrum is resolved at smaller scales and the gravity waves from high-resolution GCMs are rather realistic in comparison with satellite, airborne, and ground-based observations^7,19–21^, the spectral structures of momentum flux and forcing have not been addressed in the literature, and the mean forcing by resolved waves is still too weak in the middle and upper atmosphere. This is reflected in the excessively strong stratospheric and mesospheric zonal winds and high wind reversal altitudes^7^ and in the unrealistic periodicity of quasi-biennial oscillation (QBO)^22^ in GCM simulations, probably because the unresolved waves still have significant contribution to the global momentum budget^23^. In the case of the Whole Atmosphere Community Climate Model (WACCM) with a horizontal resolution of ~25 km^7^, the model could effectively resolve waves with horizontal wavelengths down to ~250 km. Since gravity waves can still be prominent down to small meso-beta scales (~20 km) according to results from regional cloud-resolving model with horizontal resolution of ~1 km^24^ and recent observations^25^, WACCM fails to account for the forcing by waves with horizontal wavelengths between 250 and ~20 km (either severely damped by numerical dissipation or unresolved). To quantify this missing force remains a challenge and is the objective of the current study.

Quantification of gravity wave forcing has been the main objective of various parameterization schemes^26,27^, which with careful tuning are needed for GCMs to attain the observed wind and temperature structures, especially in the stratosphere, mesosphere, and lower thermosphere. It is also determined, however, that the gravity wave parameterization schemes are a main source of model uncertainty and bias in the middle and upper atmosphere^28^. The bias limits the model capability not only for the middle and upper atmosphere but also for the troposphere, since the downward influence of the former on the latter is increasingly appreciated^29–32^. Problems of parameterization schemes include over-simplification and unphysical assumptions employed with regard to the source, propagation, and impact of gravity waves. Parameter settings in these schemes may also require adjustment for different models and model configurations. When the model resolution increases and the gravity waves are partially resolved (noted as the gray zone^33^), it becomes particularly challenging to maintain the physical consistency between the resolved portion of the gravity waves and the parameterized ones—an issue a scale-aware parameterization scheme needs to address.

This study demonstrates that, by using scale invariance as a constraint for the statistical distribution of waves over zonal scales down to ~20 km, as evidenced by previous studies^1,8^, one can estimate the mean momentum flux and forcing by the unresolved (including the under-resolved) gravity waves. The validity of the method is tested in two ways: First by applying the method over spectral ranges where these waves are still properly resolved, so that the actual forcing by these waves can be calculated to verify the deduced forcing; then by comparing the deduced forcing over the unresolved scales with the parameterized gravity wave forcing, which is obtained in bringing the simulated climatological wind in agreement with the observed. It is shown that the small-scale forcing has leading order effect on the mean flow. Scale invariance thus makes it possible to obtain a more complete description of the wave forcing when only part of the wave spectrum is resolved by the model.

## Results

### Spectral structures of horizontal and vertical winds

**Fig. 1:**
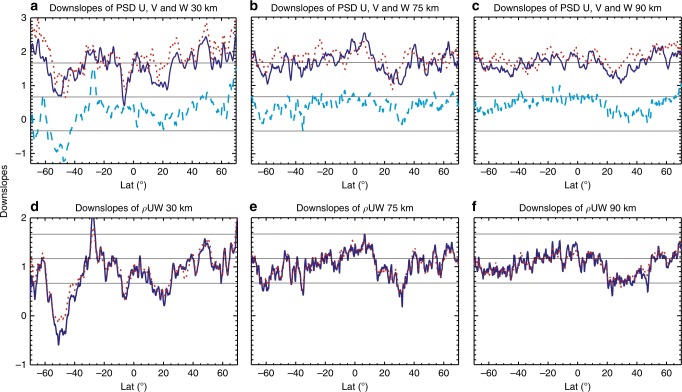
Spectral slopes of winds and momentum fluxes and their latitudinal and height dependence. **a**–**c** Downslope values (positive: downslope) of the power spectral densities of zonal (solid lines), meridional (dotted lines), and vertical winds (dashed lines) for **a** 30 km, **b** 75 km, and **c** 90 km. The thin horizontal lines in **a**–**c** denote downslope values of 5/3, 2/3 and upslope value of 1/3 (from top to bottom). **d**–**f** Similar to **a**–**c** but for downslope values of zonal momentum flux spectra (solid lines). Dotted lines in **d**–**f** are the sum of downslope values of U and W spectra. The thin horizontal lines in **d**–**f** denote downslope values of 5/3, 7/6, and 2/3 (from top to bottom)

The spectral slopes of the zonal wavenumber power spectral densities (PSDs) of zonal, meridional, and vertical winds from WACCM simulations are calculated first. Figure 1a–c show the spectral downslope values (denoted by *α*) at 30, 75, and 90 km altitudes (corresponding to the stratosphere, mesosphere, and mesopause) for latitudes up to 70°. The slopes of both horizontal wind components (*u* and *v*) vary around −5/3 (i.e., downslope value of 5/3), consistent with the Nastrom–Gage spectrum^1^. From the figure, it is seen that the variability of the slopes decreases with altitude: −*α*_*u*,*v*_ are between −0.5 and −3 at 30 km, −1 and −2.6 at 75 km, and −1.1 and −2.3 at 90 km. The vertical wind spectra are found to be rather flat, in agreement with observations^8^, with −*α*_*w*_ between −2/3 and 1/3 at most latitudes. A notable exception is at mid-high southern latitudes (~40–60°S) at 30 km (and up to the lower mesosphere), where the spectra have large upslopes, between 1/3 and 1. The significance of this departure will be discussed in later sections.

The zonal wavenumber spectrum of the vertical wind is related to the horizontal wind spectra through the continuity equation, which under Fourier transform in the zonal direction becomes

1 $$V_y + W_z = - {\mathrm{i}}kU \sim - a_u{\mathrm{i}}k^{ - \alpha _u/2 + 1}$$

where *k* is the zonal wavenumber; *U*, *V*, *W* are zonal, meridional, and vertical winds in zonal wavenumber space, with the subscripts of *y* and *z* denoting partial derivatives in meridional and vertical direction, respectively; and *a*_*u*_ is the complex power-law coefficient. According to Eq. 1, the PSD of *V*_*y*_ + *W*_*z*_ is equal to that of *U*_*x*_, thus has an upslope −*α*_*u*_ + 2. Model results indicate that the spectral shapes of *V*_*y*_, *W*_*z*_, and *W* are similar ($\alpha _{vy} \sim \alpha _{wz} \sim \alpha _w$), and they are between −*α*_*u*_ + 2 and −*α*_*u*_ + 1 (Supplementary Fig. 1). With both *α*_*u*_ and *α*_*v*_ (downslope values) around 5/3, the PSD slopes of the vertical wind are between −2/3 and 1/3 at most latitudes and altitudes, as shown in Fig. 1a–c.

### Spectral structures of wave momentum flux and forcing

Vertical winds are generally smaller than the horizontal winds in the atmosphere and thus may not contribute significantly to the total KE. However, they are critical for the vertical flux of the horizontal momentum associated with waves. In particular, the shallow spectral slopes have important implications for the momentum flux by small-scale waves. The spectral structure of the momentum flux can be quantified from the cospectra of horizontal and vertical winds, $\rho {\mathrm{Re}}(UW^ \ast )$ (vertical flux of zonal momentum, Re denotes real part) and $\rho {\mathrm{Re}}(VW^ \ast )$ (vertical flux of meridional momentum). As seen from examples in the “Methods” section, the momentum flux spectra follow power-law in the resolved range above zonal wavenumber 10. Assuming one dominant power law for each wind component, then

2 $$\tau _{uw} \equiv \rho {\mathrm{Re}}(UW^ \ast ) \sim {\mathrm{Re}}(a_ua_w^ \ast )k^{ - (\alpha _u + \alpha _w)/2}$$

3 $$\tau _{vw} \equiv \rho {\mathrm{Re}}(VW^ \ast ) \sim {\mathrm{Re}}(a_va_w^ \ast )k^{ - (\alpha _v + \alpha _w)/2}$$

and the momentum flux spectra should have slope values between −*α*_*u*,*v*_ + 1 and −*α*_*u*,*v*_ + 1/2. These values are generally consistent with the numerical results in Fig. 1d–f, which show the slopes of zonal momentum flux spectra (absolute values) calculated from the model results (solid lines), and along with it the sum of the spectral slopes of *U* and *W* (dotted lines) for comparison. The momentum flux spectral slopes vary between −2/3 and −7/6 at most latitudes and altitudes. Major departure is found again at southern mid- to high-latitudes stratosphere and lower mesosphere, while minor departures are at lower to middle latitudes at increasing altitudes. In these instances of departures, the downslope values drop below 2/3, and even below 0 (thus becoming upslope) at southern mid- to high-latitudes, indicating the significance of small-scale waves.

**Fig. 2:**
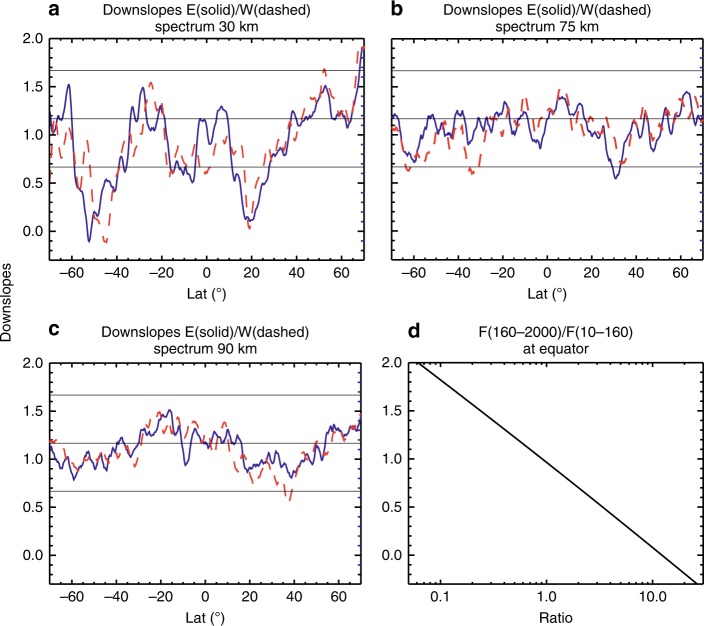
Spectral slopes of east and westward forcing and the ratio between the zonal forcing by unresolved and resolved scales. Downslope values for the zonal spectra of eastward (solid) and westward (dashed) forcing for **a** 30 km, **b** 75 km, and **c** 90 km. The thin horizontal lines denote downslope values of 5/3, 7/6, and 2/3 (from top to bottom). **d** The ratio between zonal forcing by waves with zonal wavenumbers between 160 and 2000 (zonal wavelengths 250–20 km at the equator) and by waves with zonal wavenumbers between 10 and 160 (zonal wavelengths 4000–250 km at the equator) and its dependence on the downslope values of the forcing spectra

**Fig. 3:**
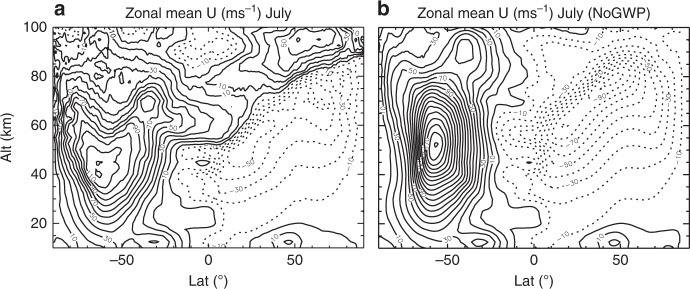
Zonal mean zonal wind from Whole Atmosphere Community Climate Model simulations. Zonal mean zonal wind **a** with and **b** without parameterized gravity wave forcing. Contour intervals: 10 ms^−1^ (solid: eastward)

The zonal forcing spectrum is calculated from the vertical divergence of the zonal momentum flux spectrum. It is then separated into spectra of eastward and westward forcing for this analysis. Both spectra display power-law scaling, though their slope values can be quite different (see “Methods”). The latitude- and height-dependent spectral slopes are shown in Fig. 2a–c. The spectral shape of the zonal forcing is similar to that of the momentum flux, with slopes of both the eastward and westward forcing between −2/3 and −7/6 at most latitudes and altitudes. Like the momentum flux spectra, major and minor departures are found at southern mid-high latitudes at 30 km up into the lower mesosphere and various latitudes/altitudes in the northern hemisphere (NH). In the former case, the westward forcing spectra have downslopes that are shallower or even upslopes that are steeper than the eastward forcing spectra (40–50°S at 30 km, and over broader latitudes in the lower mesosphere, as can be seen in “Methods”), underscoring a more profound role of westward propagating, small-scale waves. By examining the zonal mean zonal wind (Fig. 3a), it is seen that the latitudes/altitudes of the aforementioned major departure from the nominal slope values are where the simulated winter jet in the stratosphere/mesosphere is much stronger than the observed climatology^34^, presumably due to the weak westward forcing in the model. This is a known bias in many global chemistry–climate models^35–37^. In the NH where the slopes show minor departures from the nominal values in the mesosphere (Fig. 2 and also see “Methods”), the downslopes of the eastward forcing spectra are shallower than those of the westward forcing spectra, indicating the prominent role of eastward propagating, small-scale waves. These locations generally coincide with where the westward wind is strong in the summer stratosphere (low latitudes) and mesosphere (midlatitudes).

It is noted that slope values shown in Figs. 1 and 2 are averages of hourly spectra over 1 day. While the slope values vary from day to day, the features discussed above are robust and are shared by the daily average values and the mean values over a week (see “Methods”).

### Scale dependence of wave forcing

By assuming scale invariance in their spectral distribution, the zonal momentum flux and forcing by gravity waves over a zonal wavenumber range, from *k*_<_ to *k*_>_ (*k*_*<*_ < *k*_>_), can be quantified by integrating their respective spectra over this range:

4 $$\begin{array}{*{20}{l}} {F_{{\mathrm{E}},{\mathrm{W}}}^{ < > }} \hfill & = \hfill & {\int_{k_ < }^{k_ > } {\kern 1pt} f_{{\mathrm{E}},{\mathrm{W}}}k^{ - \alpha _{{\mathrm{E}},{\mathrm{W}}}}\mathrm dk} \hfill \\ {} \hfill & = \hfill & {\left\{ {\begin{array}{*{20}{l}} {f_{{\mathrm{E}},{\mathrm{W}}}(k_ > ^{1 - \alpha _{{\mathrm{E}},{\mathrm{W}}}} - k_ < ^{1 - \alpha _{{\mathrm{E}},{\mathrm{W}}}})/(1 - \alpha _{{\mathrm{E}},{\mathrm{W}}})} \hfill & {{\mathrm{if}}\,\alpha _{{\mathrm{E}},{\mathrm{W}}} \ne 1{\kern 1pt} } \hfill \\ {f_{{\mathrm{E}},{\mathrm{W}}}{\mathrm{ln}}(k_ > /k_ < )} \hfill & {{\mathrm{if}}\,\alpha _{{\mathrm{E}},{\mathrm{W}}} = 1} \hfill \end{array}} \right.} \hfill \end{array}$$

*F* in the equation refers to forcing or momentum flux with the subscripts E/W for Eastward/Westward components. The superscript <> denotes integration from a small wavenumber cutoff *k*_<_ to a large wavenumber cutoff *k*_>_. Equation 4 is used to further elucidate the forcing by smaller scale waves, with zonal wavenumber larger than *k*_|_ (denoted as *F*^|>^) as compared to larger scales (*F*^<|^):

5 $${F_{{\mathrm{E}},{\mathrm{W}}}^{| > }/F_{{\mathrm{E}},{\mathrm{W}}}^{ < |} = \left\{ {\begin{array}{*{20}{l}} {((k_ > /k_|)^{1 - \alpha _{{\mathrm{E}},{\mathrm{W}}}} - 1)/(1 - (k_ < /k_|)^{1 - \alpha _{{\mathrm{E}},{\mathrm{W}}}})} \hfill & {{\mathrm{if}}\,\alpha _{{\mathrm{E}},{\mathrm{W}}} \ne 1{\kern 1pt} } \hfill \\ {{\mathrm{ln}}(k_ > /k_|)/{\mathrm{ln}}(k_|/k_ < )} \hfill & {{\mathrm{if}}\,\alpha _{{\mathrm{E}},{\mathrm{W}}} = 1} \hfill \end{array}} \right.}$$

WACCM simulations suggest that the zonal wavenumber PSD of zonal wind at 15 km and above follows a power law with ~−5/3 starting at zonal wavenumber ~10, and down to the horizontal scale of about 250 km (corresponding to zonal wavenumber 160 at the equator), and drops sharply at higher wavenumbers due to numerical diffusion^7^. According to stratospheric measurements^8^ and a high-resolution, regional-scale numerical study^38^, the horizontal spectrum of vertical wind follows a nearly flat (former) and a slight upslope power law (latter) down to ~20 km. This corresponds to zonal wavenumber 2000 at the equator. Let *k*_<_ = 10, *k*_|_ = 160, and *k*_>_ = 2000, the ratio of the smaller- to larger-scale forcing according to Eq. 5 is plotted in Fig. 2d, and it increases rapidly as the spectrum becomes shallower: 0.15 ($\alpha _{{\mathrm{E}},{\mathrm{W}}} = 5$/3), 0.58 ($\alpha _{{\mathrm{E}},{\mathrm{W}}} = 7$/6), 0.91 ($\alpha _{{\mathrm{E}},{\mathrm{W}}} = 1$), 2.19 ($\alpha _{{\mathrm{E}},{\mathrm{W}}} = 2$/3), and 12.3 ($\alpha _{{\mathrm{E}},{\mathrm{W}}} = 0$). From Fig. 2a–c, it is seen that the downslope values *α*_E,W_ are <5/3 at almost all latitudes and altitudes, <7/6 at significant portions of latitude at 30 and 75 km, and around 7/6 at 90 km. Therefore, the smaller-scale waves can play an important, even dominant, role in forcing the general circulation in the middle and upper atmosphere. This is in contrast to the contribution to the total KE by small-scale waves, with the mean PSD slope around −5/3 (Fig. 1a–c).

**Fig. 4:**
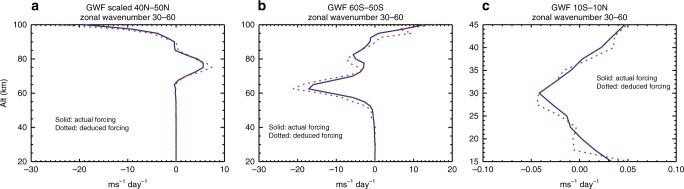
Verification of the method by comparing calculated and deduced wave forcing. Comparisons of actual forcing (solid lines) and deduced forcing (dotted lines) by waves with zonal wavenumber 30–60 for **a** 40–50°N, **b** 50–60°S, and **c** 10°S–10°N

The analysis above also provides a physically consistent method to quantify the zonal forcing by the unresolved waves in the model: the zonal forcing spectrum is determined from the vertical divergence of the zonal momentum flux spectrum, and the spectra of eastward and westward components are then used to establish their respective spectral distribution. Their spectral slopes are computed for spectral range *k*_<_ and *k*_|_ over which the power-law scaling is known to be valid in the model. By assuming the same statistical distribution for the unresolved waves, the spectral slopes obtained are used to scale the forcing of this scale range $F_{{\mathrm{E}},{\mathrm{W}}}^{ < |}$ to obtain the forcing by smaller scales $F_{{\mathrm{E}},{\mathrm{W}}}^{| > }$ according to Eq. 5. This is first tested over spectral ranges where the waves are resolved, by letting *k*_<_ = 10, *k*_|_ = 30, and *k*_>_ = 60. The deduced smaller-scale forcing *F*^|>^ is compared with the actual forcing by waves with zonal wavenumber between 30 and 60. Figure 4a–c compare the deduced forcing and actual forcing at northern midlatitudes (40–50°N), southern middle-to-high latitudes (50–60°S), and in the equatorial stratosphere, regions where the gravity wave forcing is important for driving the circulation. It is seen that the deduced forcing is in rather good agreement with the actual forcing.

**Fig. 5:**
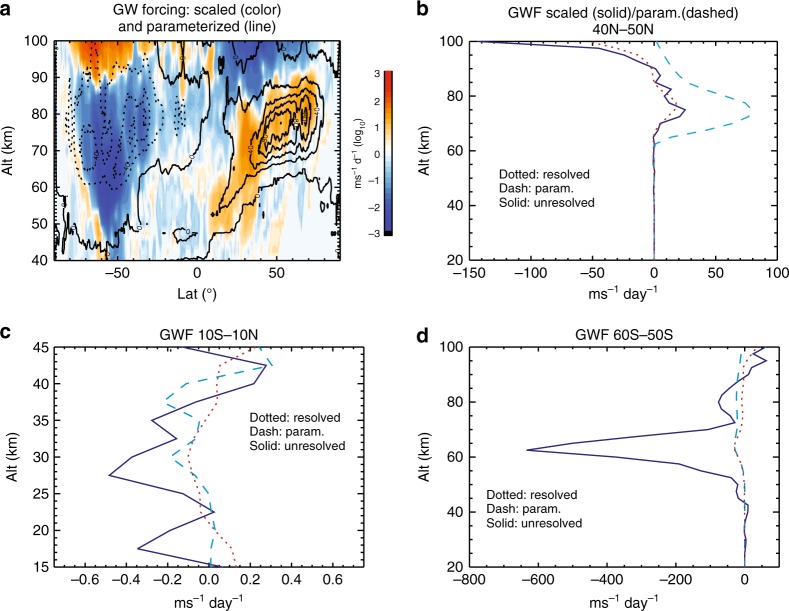
Comparing zonal forcing by resolved waves, unresolved waves, and parameterization. **a** Color contour: zonal forcing by unresolved gravity waves, deduced from the forcing by resolved gravity waves based on scale invariance. Line contours: parameterized zonal gravity wave forcing. Solid lines: eastward forcing, and contour intervals are 20 ms^−1^ day^−1^. **b**–**d** Vertical profiles of zonal forcing by resolved gravity waves (dotted lines), unresolved gravity waves (solid lines), and parameterized gravity waves (dashed lines), averaged over 40–50°N, 10°S–10°N, and 60–50°S. Base 10 logarithmic scale is used in **a**

The forcing by unresolved waves is then deduced. Again set *k*_<_ to 10. *k*_|_ varies from 160 to 55 (corresponding to zonal wavelength of 250 km) and *k*_>_ from 2000 to 684, from the equator to 70° latitude. The deduced $F_{{\mathrm{E}},{\mathrm{W}}}^{| > }$ is shown in Fig. 5a (color contours). The parameterized gravity wave forcing is over-plotted (line contours) for comparison. In the northern mesosphere (70–90 km), the deduced $F_{{\mathrm{E}},{\mathrm{W}}}^{| > }$ is eastward and up to 100 ms^−1^ day^−1^. Its direction, magnitude, and structure are in general agreement with the parameterized gravity wave forcing, which is considered as a rather coarse climatological representation of the missing gravity wave force in the northern mesosphere (though with large uncertainties^28^). This agreement serves as a further check of the deduced forcing by the unresolved waves. Figure 5b provides a more detailed comparison among the profiles of the zonal forcing by the resolved waves $F_{{\mathrm{E}},{\mathrm{W}}}^{ < |}$, the deduced zonal forcing $F_{{\mathrm{E}},{\mathrm{W}}}^{| > }$, and the parameterized zonal forcing over 40–50°N. By examining 75 km altitude for this latitude range, *α*_E_ is between 0.8–1.2, shallower than *α*_W_ (0.9–1.3). The ratio $F_{\mathrm{E}}^{| > }$/$F_{\mathrm{E}}^{ < |}$ is between 0.6 and 1.6 with an average of 1.1, $F_{\mathrm{W}}^{| > }$/$F_{\mathrm{W}}^{ < |}$ is between 0.4 and 1.2 with an average of 0.9, and the average ratio of the unresolved and resolved net zonal forcing $\mathop {\sum}\nolimits_{{\mathrm{E}},{\mathrm{W}}} {\kern 1pt} F_{{\mathrm{E}},{\mathrm{W}}}^{| > }$/$\mathop {\sum}\nolimits_{{\mathrm{E}},{\mathrm{W}}} {\kern 1pt} F_{{\mathrm{E}},{\mathrm{W}}}^{ < |}$ is 1.5. It is seen that in the mesosphere the net zonal forcing by unresolved waves $\mathop {\sum}\nolimits_{{\mathrm{E}},{\mathrm{W}}} {\kern 1pt} F_{{\mathrm{E}},{\mathrm{W}}}^{| > }$ is structurally similar to the parameterized forcing near 75 km but with smaller magnitude.

In the equatorial stratosphere, the spectral slopes of momentum flux and forcing are shallow: momentum flux between 0.4 and 1.2, eastward forcing between 0.5 and 1.3, and westward forcing between 0.6 and 1 (Figs. 1 and 2). From Fig. 5c, it is seen that the deduced $F_{{\mathrm{E}},{\mathrm{W}}}^{| > }$ (averaged over 10°S–10°N) can be significantly larger than $F_{{\mathrm{E}},{\mathrm{W}}}^{ < |}$ and the parameterized forcing in the stratosphere. $F_{{\mathrm{E}},{\mathrm{W}}}^{| > }$ is mostly westward between 17 and 40 km and varies between 0 and −0.5 ms^−1^ day^−1^, with the peak westward acceleration rate at 27.5 km (~15 hPa). The average ratio $\mathop {\sum}\nolimits_{{\mathrm{E}},{\mathrm{W}}} {\kern 1pt} F_{{\mathrm{E}},{\mathrm{W}}}^{| > }$/$\mathop {\sum}\nolimits_{{\mathrm{E}},{\mathrm{W}}} {\kern 1pt} F_{{\mathrm{E}},{\mathrm{W}}}^{ < |}$ is 3.7. The magnitude and the scale dependence of the forcing obtained here is generally consistent with previous numerical calculations, from high-resolution modeling and parameterizations, of the driving of stratospheric QBO by gravity waves^22,39,40^.

In the southern hemisphere (SH), the deduced $F_{{\mathrm{E}},{\mathrm{W}}}^{| > }$ is westward and is much stronger than the parameterized gravity wave forcing, especially in the stratopause and lower mesosphere at higher latitudes: it exceeds 600 ms^−1^ day^−1^ between 50–60°S and 60–70 km, while the forcing by resolved gravity waves ($F_{{\mathrm{E}},{\mathrm{W}}}^{ < |}$) is ~30 ms^−1^ day^−1^, similar to the parameterized value (Fig. 5d). This is where the simulated eastward jet becomes much stronger than climatology and is also where the momentum flux and forcing spectra develop shallow downslopes and even upslopes as mentioned above. At 62.5 km (where the total westward forcing peaks), *α*_E_ is between 0.5 and 0.8, while *α*_W_ is between −0.1 and 0.2; $F_{\mathrm{E}}^{| > }$/$F_{\mathrm{E}}^{ < |}$ is between 1.8 and 3.6 with an average of 2.9, $F_{\mathrm{W}}^{| > }$/$F_{\mathrm{W}}^{ < |}$ is between 8 and 17 with an average of 10.8, and the average ratio $\mathop {\sum}\nolimits_{{\mathrm{E}},{\mathrm{W}}} {\kern 1pt} F_{{\mathrm{E}},{\mathrm{W}}}^{| > }$/$\mathop {\sum}\nolimits_{{\mathrm{E}},{\mathrm{W}}} {\kern 1pt} F_{{\mathrm{E}},{\mathrm{W}}}^{ < |}$ is 21.9.

### Inter-dependence of wave spectra and large-scale wind

**Fig. 6:**
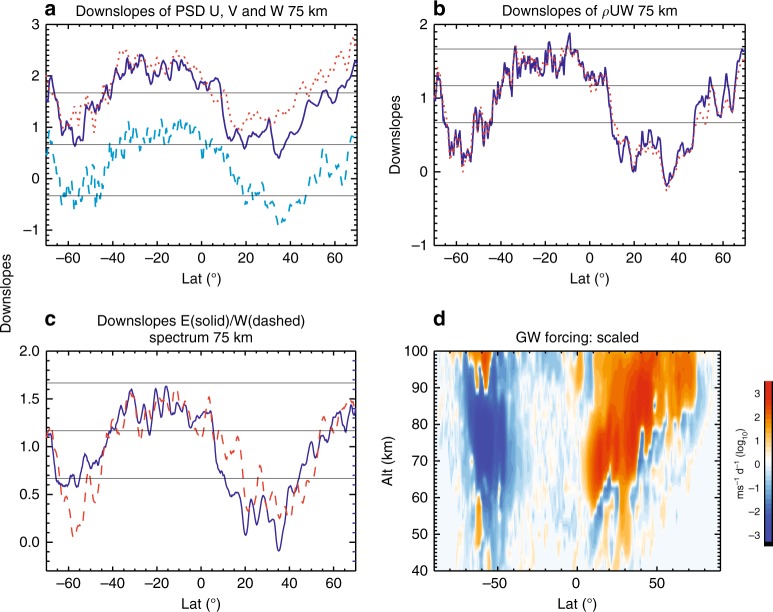
Spectral slopes and deduced zonal forcing by unresolved waves from simulation without parameterized gravity wave forcing. Downslope values of **a** power spectra of zonal, meridional, and vertical winds, **b** spectrum of vertical flux of zonal momentum, and **c** spectra of eastward and westward forcing at 75 km. **d** Zonal forcing by unresolved gravity waves deduced from the forcing by resolved gravity waves based on scale invariance. These are from the simulation without parameterized gravity wave forcing. Base 10 logarithmic scale is used in **d**

The numerical result indicates that the spectrum of the forcing opposite to the wind tends to develop a shallower downslope or slight upslope in comparison to the spectrum of waves propagating along the wind direction. This spectral asymmetry may partially result from Doppler shift of the wave system: The former wave group develops higher intrinsic frequency and larger vertical phase speed (and vertical wavelength), while the latter wave group changes oppositely, making it more vulnerable to dissipative damping and critical layer filtering. However, spectral slopes of wave forcing in both directions become shallower (Figs. 2 and 6), suggesting that small-scale waves in both directions become more significant in the region of strong, run-away jet when the wave forcing is too weak. This could be due to strong flow imbalance in the presence of strong jet, though the exact cause requires further elucidation in future studies.

According to Eq. 5 and Fig. 2d, the forcing by small-scale waves increases when the spectral downslope becomes shallower. The increasing forcing by the small-scale waves, along with the spectral asymmetry, would tend to slow down the jet. In the model, however, the increasing importance of the small-scale wave forcing is not captured beyond a certain wavenumber due to limited resolution, leading to the run-away jet. The spectral slopes of the waves are thus likely biased toward shallow values in the model results, as reflected by the shallow spectral slopes and very large deduced forcing in the stratosphere and lower mesosphere at mid–high southern latitudes in the simulation (Figs. 2 and 5). Therefore, the much shallower downslope or slight upslope from the simulation is probably an indication of missing force. In reality, the large forcing from small scales would help slow down the jet and maintain the spectral slope close to its quasi-equilibrium value. This may explain the rather robust spectral shape in observations.

The potentially important role of wave and mean flow interaction in maintaining the robust spectral structure is examined further by comparing the spectral slopes of wind PSDs and the spectra of momentum flux and forcing (shown in Figs. 1, 2 and 5) with the same quantities calculated from the WACCM simulation without gravity wave parameterization, shown in Fig. 6a–c, respectively (for 75 km). In the simulation without parameterized gravity wave forcing, the mean zonal winds in the mesosphere and stratosphere are much stronger than the climatological values (Fig. 3b), because the forcing by resolved waves are not sufficient to slow down the wind. From the comparison of the slopes, it is evident that the spectra in the simulation without parameterized gravity wave forcing have significantly shallower downslopes (or steeper upslopes) than their counterparts in WACCM with parameterized gravity wave forcing at low-to-middle latitudes in the NH and high latitudes in the SH, where the gravity wave forcing should be large (Fig. 5). Spectral asymmetry also becomes more accentuated in these regions, suggesting predominant eastward forcing in the NH and westward forcing in the SH by small-scale gravity waves. The smaller-scale forcing is deduced as discussed above (shown Fig. 6d), and it becomes very large in the mesosphere (from −3000 to 2000 ms^−1^ day^−1^), with the direction of the zonal forcing being generally consistent with that required to slow down the wind. As in the case of the SH stratosphere/lower mesosphere, the very large deduced forcing results from the flattening of the spectra in the presence of large zonal wind. It is conceivable if the deduced forcing is applied to the mean wind in the simulation, the biases of the spectral slopes (to shallow values) and the zonal forcing (to large values) would be reduced or removed.

**Fig. 7:**
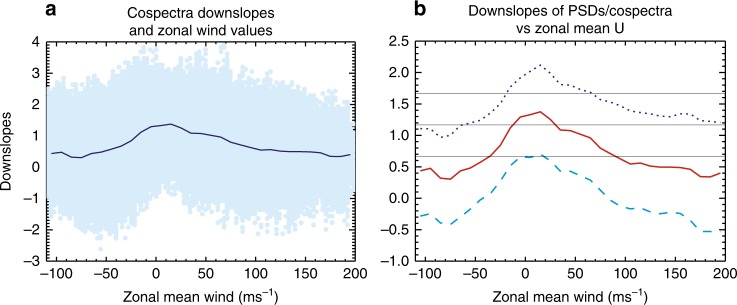
Dependence of spectral slopes on zonal mean zonal wind. **a** Scatter plot of downslope values of momentum flux spectra and zonal mean zonal wind (filled circles) from the Whole Atmosphere Community Climate Model simulation without gravity wave parameterization, derived from hourly results over model day July 6 for altitudes between 30 and 90 km and latitudes between 67°S and 67°N. The solid line are mean downslope values over intervals of 10 ms^−1^ from −110 to 200 ms^−1^. **b** Mean downslope values of the cospectra (solid line), zonal wind power spectrum density (PSD) (dotted line), and vertical wind PSD (dashed line) over intervals of 10 ms^−1^ from −110 to 200 ms^−1^. The thin horizontal lines denote downslope values of 5/3, 7/6, and 2/3 (top to bottom)

The dependence of spectral slopes on the zonal mean zonal wind is further demonstrated in Fig. 7, which shows the distribution of spectral downslope values over zonal mean zonal wind (Fig. 7a, only downslope values of momentum flux spectra) and the mean values of the spectral downslope (Fig. 7b, downslope values of zonal and vertical wind PSDs and the cospectra). In spite of their large variability, all three spectra show a clear trend of becoming flatter, with the vertical wind PSD even turning upslope, as the zonal mean zonal wind becomes stronger in both directions. This dependence is evident from the mean, as well as the maximum and minimum downslope values. Decreasing rates of downslope values appear to be larger with increasing westward wind.

## Discussion

The primary goal of this study is to examine the zonal wavenumber spectra of momentum flux and forcing, their implication for global momentum budget, and the interdependence of the spectra and the mean flow. The analysis suggests that the momentum flux and forcing spectra display scale invariance at meso-alpha scales (horizontal scales down to ~200 km) and have shallow slopes. By assuming the same statistical distribution at unresolved meso-beta scales (20–200 km), one can quantify the mean zonal forcing by the unresolved waves. Since the momentum flux and forcing spectra have shallow slopes, the mean forcing by the small-scale waves is found to have leading order effect on the mean wind. Such effects have been speculated, but the direct quantification presented here demonstrates their fundamental importance and explains the lack of convergence of mean wave forcing with the increasing spatial resolution. The calculated mean forcing is corroborated by the parameterized gravity wave forcing.

**Fig. 8:**
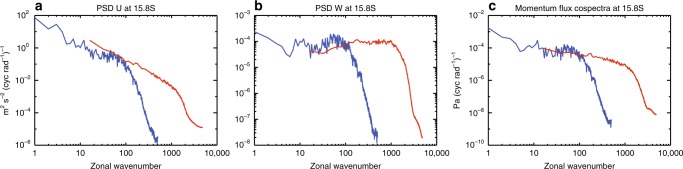
Wind power spectra and momentum flux spectra from Whole Atmosphere Community Climate Model (WACCM) and Weather Research and Forecast (WRF) simulations. Daily average of hourly power spectrum densities (PSDs) of zonal and vertical winds (**a**, **b**), and of hourly momentum flux spectra (**c**) at 15.8°S from WACCM (blue) and WRF model (red) simulations (model day: February 5). The magnitude of the vertical winds from the WRF simulation is generally larger than those in WACCM, and the red line in **b** is PSD_*W*_(WRF)/7

Previous observational and numerical studies have deduced momentum flux spectrum as related to tropospheric sources (e.g. deep convection)^38,41–43^ but have not systematically examined the scale invariance of the cospectrum. To further investigate the scale invariance from global to meso-beta scales, results from two model simulations are analyzed: a high-resolution WACCM simulation for global to meso-alpha scales and a Weather Research and Forecast (WRF) simulation for meso-alpha and meso-beta scales. The WRF simulation has a horizontal resolution of 4 km, and its initial and boundary condition is specified by the WACCM simulation every 6 h. Detailed information of this WRF/WACCM simulation can be found in ref. ^44^ and is briefly summarized in “Methods”. Figure 8 shows that the zonal and vertical winds PSDs and the momentum flux spectrum from the WRF simulation follow scale invariance down to zonal wavenumber ~1000 (40 km), and their spectral slopes are similar to those from the WACCM simulation between zonal wavenumbers 10 and 100. In particular, the vertical wind PSD from the WRF/WACCM simulation is flat and the momentum flux spectrum is shallow. These results therefore support the scale invariance assumption of momentum flux spectra throughout meso-alpha and meso-beta scales. While direct verification of this assumption with regard to the scale-invariance of momentum flux will rely on future measurements with high horizontal resolution and direct numerical simulations across the full scale range from global to meso-beta, existing observations and numerical simulations already provide partial and indirect support: the KE spectrum follows a −5/3 slope between 2.6 and 300–400 km in the troposphere and lower stratosphere^1^; in a global model with 3.5-km horizontal resolution^5^, the total KE spectrum follows a shallow slope (slightly shallower than −5/3) between zonal wavenumber of 30 and 1000 at 200 hPa between 40–50°N; and in another global modeling study with 3-km horizontal resolution^6^, the KE spectrum of the divergence mode has a similar shallow slope between horizontal wavelengths of 20 and 6000 km in the lower stratosphere. The observed vertical wind spectrum is flat between horizontal wavelength of 20 and 100 km (the maximum resolvable scale for that study) in the lower stratosphere^8^, and the simulated vertical wind spectra are flat for zonal wavenumber 10–1000 at 200 hPa between 40°N and 50°N in one study^5^ and display upslope between horizontal wavelengths of 30 and 1000 km in the lower stratosphere in the other^6^. These are similar to the vertical wind spectra in the meso-alpha range (200–2000 km) in the WACCM simulation. Gravity waves at meso-gamma range (2–20 km) may still be prominent according to high-resolution regional simulations^24,38^. However, they are more likely to be trapped by larger-scale waves^38^. Further, the measured vertical wind PSD drops quickly beyond horizontal wavelength of ~20 km^8^, implicating their diminishing contribution to the global momentum budget.

This study provides a conceptual strategy for scale-aware sub-grid scale parameterization, though designing a parameterization scheme is out of the scope of this study. Building a practical parameterization requires further investigation, development, and testing to address important scientific and technical issues. While daily and zonal averages are conducted here for studying the mean effects, it will be worth investigating if regional effects and wave intermittency^45,46^ could be incorporated to quantify momentum flux and forcing over smaller spatial and temporal scales. The accuracy of the spectral extrapolation will depend on the resolved scales and the small wavelength cutoff. Twenty kilometers is used in this study, based on lower stratospheric measurements^8^ and numerical simulations of the troposphere/lower stratosphere^5,6,38^. Future studies should determine whether this cutoff value is applicable at higher altitudes. It is also necessary to further investigate the horizontal wavelength where the KE spectrum transition from the steeper slope to the shallower slope and its dependence on altitude (see discussions in “Methods”).

Previous attempts were made to parameterize gravity wave forcing using the KE spectrum in the vertical direction (with a universal slope, nominally −3)^47–49^. Frequency and/or horizontal wavenumber spectra of energy and momentum fluxes were then modeled using gravity wave polarization and dispersion relations. The current work differs from these previous studies by examining the scale invariance of the momentum flux spectra and forcing spectra, especially the implication of the flatness of the spectra. Although the mesoscale motion that contributes to the momentum flux and forcing are thought to be associated with gravity waves, the calculation and analysis presented here do not invoke any properties specific to gravity waves (e.g., dispersion or polarization relations) or make a priori assumptions with regard to spectral slopes or amplitudes. The spectral shape therefore varies spatially and temporally, which proves to be important in exposing the inter-dependence of wave spectra and large-scale flow.

Resolving all mesoscale waves in a global model would require a horizontal resolution of ~2 km or finer. Assuming a corresponding increase of the vertical resolution to ~100 m and decrease of time stepping to keep numerical stability, the computing requirement would be 10^3^–10^4^ times that of the current state-of-the-art high-resolution GCMs, such as the one used for this study (~25 km horizontal resolution, 500 m vertical resolution). It is not yet practical to make climate (especially whole atmosphere chemistry–climate) or large ensemble runs with such resolutions. The method proposed here can potentially be used to address this need. More broadly, the method may also be applied to evaluate effects by processes at unresolvable scales in other systems, given the ubiquity of scale invariance in nature and the common need for scale-aware parameterization of sub-grid processes in numerical models.

## Methods

### Numerical models

National Center for Atmospheric Research (NCAR) WACCM simulations are used in this study. WACCM is one of the atmosphere components of the NCAR Community Earth System Model (CESM), with the vertical domain extending to 5.9 × 10^−6^ hPa (~145 km). For the current study, the spectral element dynamical core is used on a cubed sphere with a quasi-uniform horizontal resolution of ~25 km and a 0.1 scale height vertical resolution >40 hPa (and higher below). Details of the model can be found in ref. ^7^ and references therein. Simulations with and without gravity wave parameterization scheme^7,50^, both for the month of July, are used for the current study. The gravity wave parameterization scheme used in the standard WACCM configuration^51^ has been adjusted^50^ to obtain realistic mean wind and temperature structures.

The Advanced Research WRF^52^ simulation employed here for the spectral analysis is described in detail in ref. ^44^, and briefly summarized here: WRF horizontal domain is between 179°E–157°W and 13°–31°S, with horizontal resolution of 4 km. The model vertical domain extends from the surface to 1 hPa (~46 km) with 138 levels, and the top 10 km is a damping layer. The initial and lateral boundary conditions of the WRF simulation are specified by the high-resolution WACCM output for the time period of 00 UTC 4 February to 12 UTC 6 February^7^. The WACCM model set-up is the same as the one used for the current study. WRF simulation results for 5 February are used for the spectral analysis presented in Fig. 8.

### Calculation of a power-law slope

The following method is used to calculate the slope of a spectrum with power-law scaling, $f(k) = ak^{ - \alpha }$. Choosing 3 wavenumbers, *k*_1_, *k*_2_, and *k*_3_ in ascending order, within the range where the power law is valid, the ratio of the spectral integrations over *k*_1_ and *k*_2_ and over *k*_2_ and *k*_3_ is:

6 $$r(k_1,k_2,k_3) \equiv \frac{{{\int}_{k_2}^{k_3} f(k){\mathrm{d}}k}}{{{\int}_{k_1}^{k_2} f(k){\mathrm{d}}k}} = \left\{ {\begin{array}{*{20}{l}} {\frac{{(k_3/k_2)^{1 - \alpha } - 1}}{{1 - (k_1/k_2)^{1 - \alpha }}}} \hfill & {{\mathrm{if}}\,\alpha \, \ne \, 1{\kern 1pt} } \hfill \\ {\frac{{{\mathrm{ln}}(k_3/k_2)}}{{{\mathrm{ln}}(k_2/k_1)}}} \hfill & {{\mathrm{if}}\,\alpha = 1} \hfill \end{array}} \right.$$

It is thus possible to determine the index *α* from this ratio. In WACCM simulations, the shallow slope extends to zonal wavenumber ~10 above 15 km altitude, as noted earlier. It is thus convenient to choose $k_1 = 10$, $k_2 = 20$, $k_3 = 40$ and then

7 $$\alpha = 1 - {\mathrm{ln}}{\kern 1pt} r(10,20,40)/{\mathrm{ln}}{\kern 1pt} 2$$

and Eq. 7 is valid for all *α* values. It is noted that with the quasi-uniform horizontal resolution of ~25 km, WACCM simulation can effectively resolve waves with horizontal wavelength of 200–250 km^7^, and the power-law scaling discussed in this work is valid at zonal wavenumber 55 equator-ward of 70° latitude. The spectral results discussed in this study are daily averages of hourly analysis.

### Spectra of divergence and rotational modes

Previous studies^2,6^ suggested that the horizontal wavelength where the KE spectrum transitions from the steeper slope to the shallower slope depends on the relative magnitudes of the rotational and divergence modes, with the latter associated closely with gravity waves. However, the vertical component of vorticity (Ω) of a gravity wave is only approximately zero when the wave intrinsic frequency is much higher than the inertial frequency ($\omega \gg f$), since

8 $${\mathrm{\Omega }} = {\mathrm{i}}\frac{{f(k^2 + l^2)}}{{{\mathrm{i}}\omega l + fk}}V$$

according to the dispersion relation of a gravity wave^53^. Therefore, both divergence and rotational modes include gravity waves, which was also noted by Koshyk et al.^2^, and the use of Helmholtz–Hodge decomposition of horizontal winds to isolate gravity waves is a valid approximation only if these are high-frequency gravity waves. By applying the decomposition to WACCM simulation results at different altitude, it is found that both divergence and rotational modes display the shallower slope at increasing spherical wavenumber ranges at higher altitudes and that the magnitudes of the two within the shallow spectral range become quite comparable (Supplementary Fig. 2), consistent with previous model results^2^. The transition wavenumber from the steep slope to the shallower slope could still be related to the relative dominance of gravity waves, but it may not be optimally quantified by comparing divergence and rotational modes.

### Spectral structures and variability of spectral slopes

**Fig. 9:**
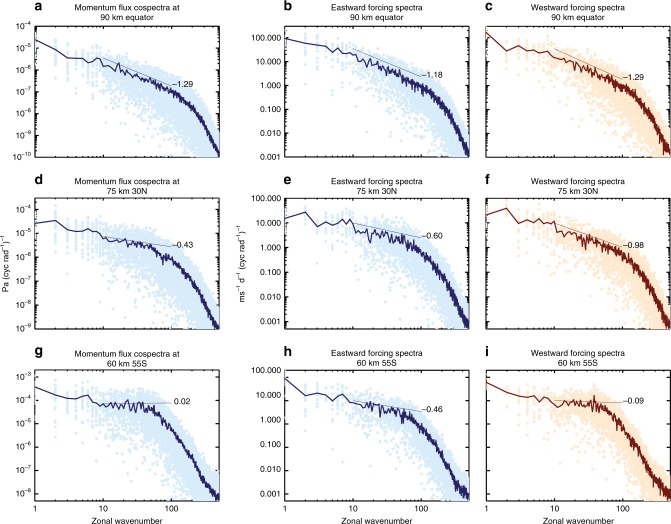
Zonal wavenumber spectra of momentum flux and eastward and westward forcing. Cospectra of zonal and vertical winds (**a**, **d**, **g**) and spectra of the eastward (**b**, **e**, **h**) and westward (**c**, **f**, **i**) forcing at 60 km/55°S (**g**–**i**), 75 km/55°N (**d**–**f**), and 90 km/equator (**a**–**c**). Both hourly (filled circles) and daily average values (line plots) are plotted. Thin straight lines indicate the slope values calculated from Eq. 7

Figure 9 shows spectra of the vertical flux of zonal momentum and the spectra of eastward and westward forcing (both hourly values and their daily averages are plotted) at several altitudes/latitudes. It is evident that they all follow power law up to zonal wavenumbers where numerical dissipation becomes important. The slope values in the plots are calculated from the daily average spectra according to Eq. 7. The difference between the spectral slopes of eastward and westward forcing is clearly seen at 60 km/55°S, where the scaled forcing peaks. It is seen that the hourly spectra vary quite significantly within a day.

**Fig. 10:**
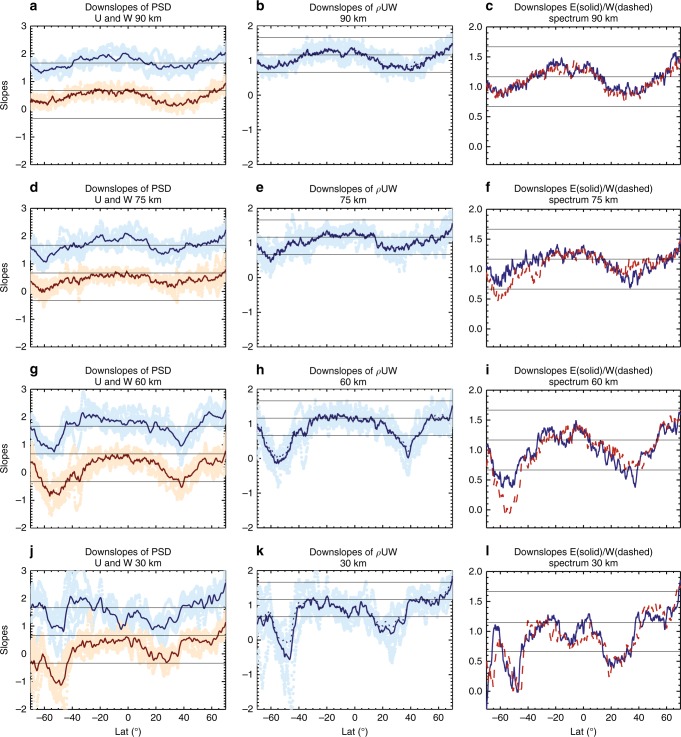
Daily and weekly averages of spectral slopes of winds, momentum fluxes, and zonal forcing. Daily (filled circles) and weekly (line plots) averages of downslope values of zonal and vertical wind power spectrum densities (PSDs) (**a**, **d**, **g**, **j**, blue: zonal wind; orange: vertical wind) and momentum flux spectra (**b**, **e**, **h**, **k**, dotted lines are the sum of downslope values of *U* and *W* spectra). **c**, **f**, **i**, **l** Weekly averages of downslope values of eastward (solid) and westward (dashed) forcing (daily values not shown for figure clarify). Values are shown at altitudes **a**–**c** 90 km, **d**–**f** 75 km, **g**–**i** 60 km, and **j**–**l** 30 km

The variability of the daily values is also examined. Figure 10 shows the range of slope variability for the wind PSDs, momentum flux spectra, and zonal forcing spectra at different altitudes. The weekly average values are similar to those in Figs. 1 and 2.

## Supplementary information

Supplementary Information

## Data Availability

Outputs from model simulations used in this study are archived on the NCAR High Performance Storage System and are accessible through Earth System Grid (https://www.earthsystemgrid.org/).

## Code availability

NCAR CESM/WACCM and Advanced Research WRF are open-source community models. WACCM source code is available at 10.5065/D67H1H0V. WRF source code is available at http://www2.mmm.ucar.edu/wrf/users/download/get_source.html.

## References

[1] Nastrom GD, Gage KS (1985). A climatology of atmospheric wavenumber spectra of wind and temperature observed by commercial aircraft. J. Atmos. Sci..

[2] Koshyk JN, Boville BA, Hamilton K, Manzini E, Shibata K (1999). Kinetic energy spectrum of horizontal motions in middle-atmosphere models. J. Geophys. Res..

[3] Koshyk JN, Hamilton K (2001). The horizontal kinetic energy spectrum and spectral budget simulated by a high-resolution troposphere–stratosphere–mesosphere GCM. J. Atmos. Sci..

[4] Takahashi YO, Hamilton K, Ohfuchi W (2006). Explicit global simulation of the mesoscale spectrum of atmospheric motions. Geophys. Res. Lett..

[5] Terasaki K, Tanaka HL, Satoh M (2009). Characteristics of the kinetic energy spectrum of nicam model atmosphere. SOLA.

[6] Skamarock WC, Park S-H, Klemp JB, Snyder C (2014). Atmospheric kinetic energy spectra from global high-resolution nonhydrostatic simulations. J. Atmos. Sci..

[7] Liu H-L (2014). Gravity waves simulated by high-resolution Whole Atmosphere Community Climate Model. Geophys. Res. Lett..

[8] Bacmeister JT (1996). Stratospheric horizontal wavenumber spectra of winds, potential temperature, and atmospheric tracers observed by high-altitude aircraft. J. Geophys. Res. Atmospheres.

[9] Fritts David C., Smith Ronald B., Taylor Michael J., Doyle James D., Eckermann Stephen D., Dörnbrack Andreas, Rapp Markus, Williams Bifford P., Pautet P.-Dominique, Bossert Katrina, Criddle Neal R., Reynolds Carolyn A., Reinecke P. Alex, Uddstrom Michael, Revell Michael J., Turner Richard, Kaifler Bernd, Wagner Johannes S., Mixa Tyler, Kruse Christopher G., Nugent Alison D., Watson Campbell D., Gisinger Sonja, Smith Steven M., Lieberman Ruth S., Laughman Brian, Moore James J., Brown William O., Haggerty Julie A., Rockwell Alison, Stossmeister Gregory J., Williams Steven F., Hernandez Gonzalo, Murphy Damian J., Klekociuk Andrew R., Reid Iain M., Ma Jun (2016). The Deep Propagating Gravity Wave Experiment (DEEPWAVE): An Airborne and Ground-Based Exploration of Gravity Wave Propagation and Effects from Their Sources throughout the Lower and Middle Atmosphere. Bulletin of the American Meteorological Society.

[10] Shepherd TG, Koshyk JN, Ngan K (2000). On the nature of large-scale mixing in the stratosphere and mixing. J. Geophys. Res..

[11] VanZandt TE (1982). A universal spectrum buoyancy waves in the atmosphere. Geophys. Res. Lett..

[12] Farge M, Sadourny R (1989). Wave-vortex dynamics in rotating shallow water. J. Fluid Mech..

[13] Yuan L, Hamilton K (1994). Equilibrium dynamics in a forced-dissipative F-plane shallow-water system. J. Fluid Mech..

[14] Tulloch R, Smith KS (2006). A theory for the atmospheric energy spectrum: depth-limited temperature anomalies at the tropopause. Proc. Natl Acad. Sci..

[15] Lilly DK (1983). Stratified turbulence and the mesoscale variability of the atmosphere. J. Atmos. Sci..

[16] Gage KS, Nastrom GD (1986). Theoretical interpretation of atmospheric wavenumber spectra of wind and temperature observed by commercial aircraft during gasp. J. Atmos. Sci..

[17] Vallis GK, Shutts GJ, Gray MEB (1997). Balanced mesoscale motion and stratified turbulence forced by convection. Q. J. R. Meteorol. Soc..

[18] Hamilton K, Takahashi YO, Ohfuchi W (2008). Mesoscale spectrum of atmospheric motions investigated in a very fine resolution global general circulation model. J. Geophys. Res. Atmospheres.

[19] Liu H-L (2016). Variability and predictability of the space environment as related to lower atmosphere forcing. Space Weather.

[20] Fritts David C., Smith Ronald B., Taylor Michael J., Doyle James D., Eckermann Stephen D., Dörnbrack Andreas, Rapp Markus, Williams Bifford P., Pautet P.-Dominique, Bossert Katrina, Criddle Neal R., Reynolds Carolyn A., Reinecke P. Alex, Uddstrom Michael, Revell Michael J., Turner Richard, Kaifler Bernd, Wagner Johannes S., Mixa Tyler, Kruse Christopher G., Nugent Alison D., Watson Campbell D., Gisinger Sonja, Smith Steven M., Lieberman Ruth S., Laughman Brian, Moore James J., Brown William O., Haggerty Julie A., Rockwell Alison, Stossmeister Gregory J., Williams Steven F., Hernandez Gonzalo, Murphy Damian J., Klekociuk Andrew R., Reid Iain M., Ma Jun (2016). The Deep Propagating Gravity Wave Experiment (DEEPWAVE): An Airborne and Ground-Based Exploration of Gravity Wave Propagation and Effects from Their Sources throughout the Lower and Middle Atmosphere. Bulletin of the American Meteorological Society.

[21] Shibuya R (2017). Quasi-12h inertia–gravity waves in the lower mesosphere observed by the pansy radar at syowa station (39.6°E, 69.0°S). Atmos. Chem. Phys..

[22] Kawatani Y (2010). The roles of equatorial trapped waves and internal inertia–gravity waves in driving the quasi-biennial oscillation. Part I: Zonal mean wave forcing. J. Atmos. Sci..

[23] Garcia Rolando R., Richter Jadwiga H. (2019). On the Momentum Budget of the Quasi-Biennial Oscillation in the Whole Atmosphere Community Climate Model. Journal of the Atmospheric Sciences.

[24] Lane TP, Reeder MJ (2001). Modelling the generation of gravity waves by a maritime continent thunderstorm. Q. J. R. Meteorol. Soc..

[25] Smith RB (2016). Stratospheric gravity wave fluxes and scales during deepwave. J. Atmos. Sci..

[26] McLandress C (1998). On the importance of gravity waves in the middle atmosphere and their parameterization in general circulation models. J. Atmos. Sol Terr. Phys..

[27] Alexander MJ (2010). Recent developments in gravity-wave effects in climate models and the global distribution of gravity-wave momentum flux from observations and models. Q. J. R. Meteorol. Soc..

[28] Pedatella NM (2014). The neutral dynamics during the 2009 sudden stratosphere warming simulated by different whole atmosphere models. J. Geophys. Res..

[29] Baldwin MP, Dunkerton TJ (2001). Stratospheric harbingers of anomalous weather regimes. Science.

[30] Collimore CC, Martin DW, Hitchman MH, Huesmann A, Waliser DE (2003). On the relationship between the QBO and tropical deep convection. J. Clim..

[31] Kidston Joseph, Scaife Adam A., Hardiman Steven C., Mitchell Daniel M., Butchart Neal, Baldwin Mark P., Gray Lesley J. (2015). Stratospheric influence on tropospheric jet streams, storm tracks and surface weather. Nature Geoscience.

[32] Yoo C, Son S (2016). Modulation of the boreal wintertime Madden–Julian oscillation by the stratospheric quasi-biennial oscillation. Geophys. Res. Lett..

[33] Vosper SB, Brown AR, Webster S (2016). Orographic drag on islands in the nwp mountain grey zone. Q. J. R. Meteorol. Soc..

[34] Swinbank, R. & Ortland, D. A. Compilation of wind data for the Upper Atmosphere Research Satellite (UARS) Reference Atmosphere Project. *J*. *Geophys*. *Res*. **108**, 4615 10.1029/2002JD003135 (2003).

[35] Austin J (2003). Uncertainties and assessments of chemistry-climate models of the stratosphere. Atmos. Chem. Phys..

[36] McLandress C, Shepherd TG, Polavarapu S, Beagley SR (2012). Is missing orographic gravity wave drag near 60°S the cause of the stratospheric zonal wind biases in Chemistry–Climate models?. J. Atmos. Sci..

[37] Garcia RR, Smith AK, Kinnison DE, de la Cámara Á, Murphy DJ (2017). Modification of the gravity wave parameterization in the whole atmosphere community climate model: Motivation and results. J. Atmos. Sci..

[38] Lane TP, Knievel JC (2005). Some effects of model resolution on simulated gravity waves generated by deep, mesoscale convection. J. Atmos. Sci..

[39] Xue X.-H., Liu H.-L., Dou X.-K. (2012). Parameterization of the inertial gravity waves and generation of the quasi-biennial oscillation. Journal of Geophysical Research: Atmospheres.

[40] Richter JH, Solomon A, Bacmeister JT (2014). On the simulation of the quasi-biennial oscillation in the community atmosphere model, version 5. J. Geophys. Res. Atmospheres.

[41] Hertzog A, Boccara G, Vincent RA, Vial F, Cocquerez P (2008). Estimation of gravity wave momentum flux and phase speeds from quasi-lagrangian stratospheric balloon flights. Part II: Results from the Vorcore campaign in Antarctica. J. Atmos. Sci..

[42] Ern, M. & Preusse, P. Gravity wave momentum flux spectra observed from satellite in the summertime subtropics: Implications for global modeling. *Geophys*. *Res*. *Lett*. **39**, L15810 10.1029/2012GL052659 (2012).

[43] Jewtoukoff V, Riwal P, Albert H (2013). Gravity waves generated by deep tropical convection: Estimates from balloon observations and mesoscale simulations. J. Geophys. Res. Atmospheres.

[44] Wu JF, Xue XH, Liu HL, Dou XK, Chen TD (2018). Assessment of the simulation of gravity waves generation by a tropical cyclone in the high-resolution WACCM and the WRF. J. Adv. Model. Earth Syst..

[45] Wright CJ, Osprey SM, Gille JC (2013). Global observations of gravity wave intermittency and its impact on the observed momentum flux morphology. J. Geophys. Res. Atmospheres.

[46] Jewtoukoff V, Hertzog A, Plougonven R, de la Cámara A, Lott F (2015). Comparison of gravity waves in the southern hemisphere derived from balloon observations and the ecmwf analyses. J. Atmos. Sci..

[47] Fritts DC, Vanzandt TE (1993). Spectral estimates of gravity wave energy and momentum fluxes. Part I: Energy dissipation, acceleration, and constraints. J. Atmos. Sci..

[48] Fritts DC, Lu W (1993). Spectral estimates of gravity wave energy and momentum fluxes, II: Parameterization of wave forcing and variability. J. Atmos. Sci..

[49] Gardner CS, Hostetler CA, Franke SJ (1993). Gravity wave models for the horizontal wave number spectra of atmospheric velocity and density fluctuations. J. Geophys. Res. Atmospheres.

[50] Liu H-L (2017). Large wind shears and their implications for diffusion in regions with enhanced static stability: the mesopause and the tropopause. J. Geophys. Res. Atmospheres.

[51] Richter JH, Sassi F, Garcia RR (2010). Toward a physically based gravity wave source parameterization in a general circulation model. J. Atmos. Sci..

[52] Skamarock, W. C. et al. A description of the advanced research WRF version 3. NCAR Technical Note NCAR/TN-475+STR (NCAR, Boulder, CO, 2008).

[53] Fritts, D. C. & Alexander, M. J. Gravity wave dynamics and effects in the middle atmosphere. *Rev*. *Geophys*. **41**, 1003 10.1029/2001RG000106 (2003).

