# Supplementary material for: Quantifying gravity wave forcing using scale invariance
Source: Nat Commun. 2019 Jun 13;10:2605. doi: 10.1038/s41467-019-10527-z (PMC6565739; doi:10.1038/s41467-019-10527-z)
Supplement: Supplementary file 1 — Supplementary Information [file 41467_2019_10527_MOESM1_ESM.pdf]

Supplementary Information

Quantifying gravity wave forcing using scale invariance

Liu

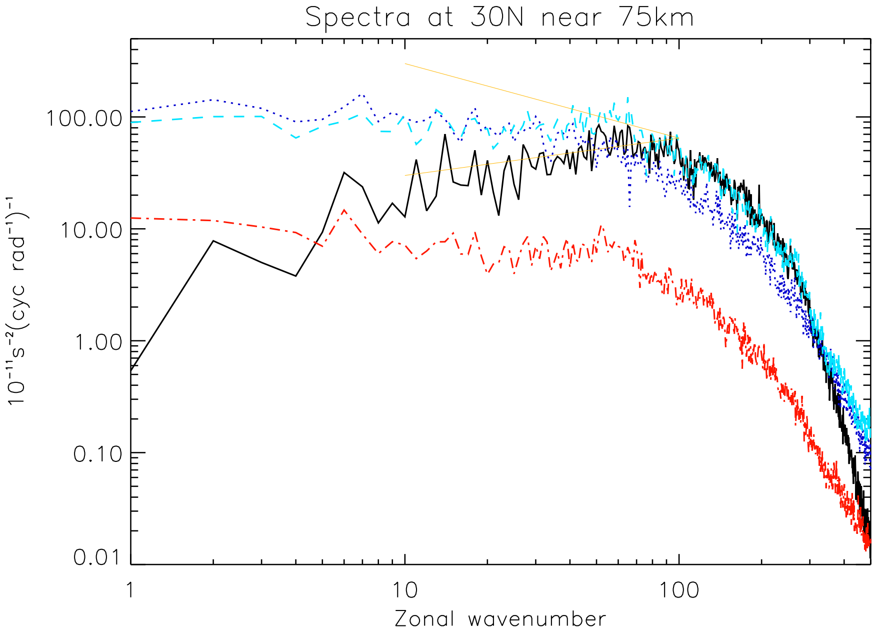

Supplementary Figure 1: **Power spectra of continuity equation terms.** Power spectra of U_x (solid line), V_y (dotted line), and W_z (dash line) at 30°N and on pressure surface 0.021 hPa (∼75 km). Power spectrum of the vertical motion is also shown (dash-dotted line) for comparison. The thin straight lines indicate slope of 1/3 (lower line) and -2/3 (upper line).

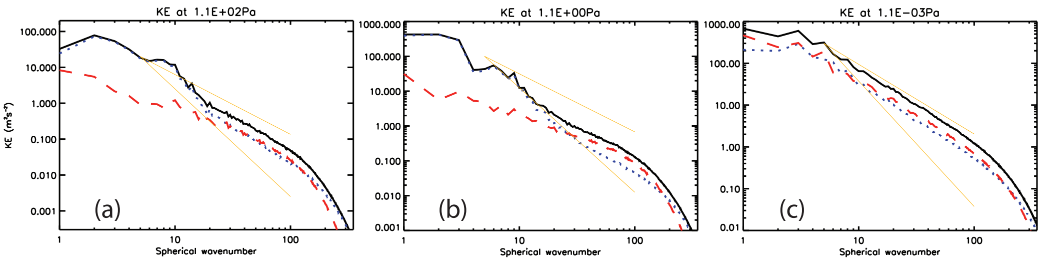

Supplementary Figure 2: **Kinetic energy spectra of divergence and rotational modes at different altitudes.** Total kinetic energy spectra (solid lines) and decomposition into divergence mode (dash lines) and rotational mode (dotted lines) at (a) 110 hPa, (b) 1.1 hPa, and (c) 1.1×10^{-3} hPa. The thin straight lines denote the -3 slope (lower line) and -5/3 slope (upper line).
